# Allo-immunisation anti-érythrocytaire chez les polytransfusés au Centre National Hospitalier Universitaire de Cotonou: à propos de 51 cas

**DOI:** 10.11604/pamj.2021.38.304.28202

**Published:** 2021-03-24

**Authors:** Tatiana Baglo, Alban Zohoun, Bruno Léopold Agboton, Jacques Vigan, Paolo Ayaka, Ludovic Anani, Awa Omar Touré Fall, Dorothée Kindé Gazard

**Affiliations:** 1 Laboratoire d’Hématologie du Centre National Hospitalier Universitaire, Cotonou, Bénin,; 2 Clinique Universitaire de Néphrologie et de Dialyse du Centre National Hospitalier Universitaire, Cotonou, Bénin,; 3 Laboratoire d´Hématologie, Hôpital Aristide Le Dantec, Dakar, Sénégal

**Keywords:** Allo-immunisation, anti-érythrocytaire, polytransfusés, Alloimmunisation, red blood cell alloimmunization, polytransfused

## Abstract

La transfusion sanguine est un acte médical qui consiste à soigner un patient avec un produit sanguin labile. Chaque transfusion de concentré globulaire expose les receveurs à un risque d´allo-immunisation anti-érythrocytaire. La recherche d´anticorps anti-érythrocytaire (RAI) permet d´assurer la sécurité immuno-hématologique des patients transfusés. Au Bénin, ce test n´est pas systématique ni lors du bilan pré-transfusionnel, ni en post transfusionnel. L'objectif de notre étude était de déterminer les allo-anticorps anti-érythrocytaires chez les polytransfusés. Pour ce, la RAI a été réalisée par le test indirect à l´antiglobuline en gel-filtration chez 51 polytransfusées dont 26 sélectionnés dans le service d´Hématologie et 25 dans le service de Néphrologie du CNHU-HKM. Après avoir phénotypé les patients allo-immunisés, la recherche des signes d´hémolyse a été effectuée chez eux. Les données cliniques et transfusionnelles des patients ont été collectées à partir des registres de transfusion et de leurs dossiers médicaux. Au terme de l'étude, la prévalence de l´allo-immunisation au sein de notre population était de 13,73%. Les anticorps identifiés avaient les spécificités suivantes: une association anti-RH1 + anti RH3, un anti LE1, puis une association anti-RH3 + anti-FY1. Les allo-anticorps étaient plus fréquents chez les patients ayant reçu plus de 15 poches de concentrés de globules rouges. Un des patients allo-immunisés présentait des signes biologiques d´hémolyse. Il n´existait pas de lien entre l´âge, le sexe, le diagnostic clinique et la survenue d´allo-immunisation anti-érythrocytaire. En conclusion, la réalisation systématique de la RAI chez les polytransfusés permettra de leur assurer une meilleure sécurité transfusionnelle au Bénin.

## Abstract (en)

Blood transfusion is a medical procedure used to treat patients with labile blood product. Each transfusion of globular concentrate exposes recipients to the risk of red blood cell alloimmunization. The test for red cell antibodies (RCA) ensures the immunohaematological safety of transfused patients. In Benin, this test is not performed in a systematic way or included either in the pre-transfusion or in the post-transfusion tests. The purpose of this study is to determine the presence of red cell antibodies among polytransfused patients. RCA was performed using indirect antiglobulin test on gel-filtration in 51 polytransfused patients including 26 selected in the Department of Hematology and 25 in the Department of Nephrology at the National Hospital and University Center of Cotonou. After phenotyping alloimmunized patients, tests for detecting signs of hemolysis were performed. Clinical data as well as those on transfusion were collected from transfusion registries and medical records. The prevalence of alloimmunization in our study population was 13.73%. The antibodies identified had the following characteristics: association of anti-RH1 and anti-RH3, anti LE1, association of anti-RH3 and anti-FY1. Alloantibodies were more frequent in patients who had received more than 15 packed red blood cells. Laboratory tests showed signs of hemolysis in one alloimmunized patient. There was no correlation between age, sex, clinical diagnosis and the occurrence of red blood cell alloimmunization. The test for red cell antibodies should be systematically performed in polytransfused patients in order to ensure better transfusion recipient safety in Benin.

## Introduction

La transfusion sanguine est un acte médical qui consiste à donner du sang ou l´un de ses dérivés prélevés chez un sujet sain appelé donneur, à un sujet malade appelé receveur [1]. C´est une thérapeutique à laquelle s´associent plusieurs complications immunologiques, infectieuses et allergiques. Malgré les efforts réalisés dans le domaine de l´immuno-hématologie, la transfusion de concentrés de globules rouges (CGR) apporte des antigènes étrangers aux receveurs. Ces antigènes érythrocytaires inconnus du receveur pourraient induire l´apparition d´allo-anticorps anti-érythrocytaires chez le receveur. Le risque de survenue d´allo-immunisation anti-érythrocytaire est donc plus important chez les sujets polytransfusés et augmentent avec le nombre de poches de concentrés de globules rouges transfusés [2]. En effet, le suivi d´une cohorte de 42 drépanocytaires polytransfusés en Côte d´Ivoire en 2013 avait révélé une prévalence d´allo-immunisation anti-érythrocytaire de 28% [3]. De même, sur 78 polytransfusés au Mali, 10,3% possédait des allo-anticorps anti-érythrocytaires [4] alors que cette prévalence était de 16,6% sur une population de 84 hémoglobinopathes polytransfusés au Maroc [5]. Cette complication a été retrouvée aussi en dehors des frontières africaines. Une étude réalisée en 2001 sur 42 autrichiens polytransfusés souffrant d´un syndrome myélodysplasique révélait une RAI positive chez 21% des patients [6].

En Inde, 9,8% des patients polytransfusés du fait d´une insuffisance rénale chronique sur 81 enquêtés, possédait des allo-anticorps anti-érythrocytaires [7]. Les anticorps impliqués au cours de ces allo-immunisations sont le plus souvent dirigés contre les antigènes des systèmes érythrocytaires RH, KEL, FY, JK et MNS [8]. Le risque d´immunisation anti-érythrocytaire du receveur dépend de plusieurs facteurs dont l´immunogénicité des antigènes de groupes sanguins apportés par les concentrés de globules rouges transfusés et inconnus du receveur, l´immunocompétence du receveur et l´occurrence de la situation d´immunisation. Au Bénin, la transfusion sanguine est réalisée dans un contexte de compatibilité dans les systèmes ABO et RH1 et la recherche d´anticorps irréguliers ne se fait pas de façon systématique au cours du bilan pré et post transfusionnel. Une telle pratique pourrait accroître les risques d´allo-immunisation anti-érythrocytaire, et engendrer des complications cliniques chez les polytransfusés tout en compromettant l´avenir obstétrical des sujets de sexe féminin. L´objectif de notre étude était donc de déterminer les allo-anticorps anti-érythrocytaires chez les polytransfusés recrutés au Centre National Hospitalier Universitaire Hubert Koutoukou Maga (CNHU HKM) de Cotonou.

## Méthodes

Il s´agissait d´une étude transversale ayant permis de recruter de façon consécutive 51 polytransfusés admis dans les services d´hématologie clinique et de néphrologie du Centre National Hospitalier et Universitaire Hubert Koutoukou Maga (CNHU HKM) de Cotonou du 1^er^ juin au 15 septembre 2016.

Les patients inclus avaient été transfusés au moins deux fois avec du concentré de globules rouges (CGR) et/ou du sang total et leur dernière transfusion datait de moins de trois mois. N´ont pas été inclus, les patients ayant une hyperprotidémie ou des agglutinines froides et ceux n´ayant pas donné leur consentement éclairé. Pour chaque patient inclus, un dépistage des agglutinines irrégulières (RAI) a été réalisé grâce à un panel de 3 cellules de groupes sanguins O phénotypés et les allo-anticorps anti-érythrocytaires dépistés ont été identifiés grâce à un autre panel de 11 cellules. Enfin le phénotypage érythrocytaire étendu et de la recherche de certains signes biologiques d´hémolyse comme la bilirubinémie et le taux sanguin de lactate déshydrogénase (LDH) avaient été réalisés chez les patients chez qui la recherche d´agglutinine irrégulière était revenue positive.

La recherche de ces allo-anticorps reposait sur la mise en œuvre d´un test indirect à l´antiglobuline polyspécifique sur un support de filtration. Les trois types d´hématies test du panel de dépistage et le plasma du patient avaient été déposés dans la chambre d´incubation au sommet de la colonne. Pendant l´incubation à +37°C, les éventuels anticorps anti-érythrocytaires présents dans le sérum du patient se fixaient sur les antigènes érythrocytaires correspondant si ceux-ci étaient présents sur les hématies tests. Le complexe ainsi formé, de taille supérieure à celle des mailles du gel était freiné lors de sa migration. Toutes les réactions ont été réalisées en milieu de basse force ionique (solution LISS) qui facilite l´accessibilité des antigènes et accroit la rapidité de la fixation des anticorps, réduisant ainsi le temps d´incubation des réactions [9].

L´intensité des réactions positives obtenues était notée de 1+ à 4+ selon le niveau de migration du complexe. Celle-ci est fonction de la quantité d´anticorps mais aussi de son avidité et de son affinité avec l´antigène [9]. En cas de dépistage positif, l´étape d´identification consistait à déterminer la spécificité du ou des anticorps présents dans le plasma du patient en confrontant la distribution des réactions positives et négatives obtenues avec la distribution des antigènes sur le panel d´hématies tests utilisées [9]. Cette analyse aboutissait à une hypothèse d´anticorps qui a été confirmé par la détermination du phénotype érythrocytaire du patient dans le système de groupe sanguin concerné. Enfin, une validation statistique de la spécificité de l´anticorps identifiée a été réalisée.

Au cours de notre étude, le phénotypage étendu avait été réalisé grâce à la technique classique en tube en milieu salin chez les patients ayant une RAI positive. Pour ce, des sérum-tests Seraclone® ont été utilisés: anti-RH1, anti-RH2, anti-RH3, anti-RH4, anti-RH5, anti-LE1, anti-LE2, anti-KEL1, anti-KEL2, anti-JK1, anti-JK2, anti- FY1, et anti-FY2. Pour la RAI, nous avons eu recours au panel de trois hématies ID-DiaCell I-II-II (Biorad®) pour le dépistage et un panel d´identification de 11 hématies ID-DiaPanel (Biorad®) pour leur identification sur carte-gel LISS-Coombs (Biorad®).

L´analyse des données a été réalisée avec le logiciel Epi Data Analysis V2.2. Le test de khi-2 a été effectué pour étudier l´association entre deux variables qualitatives afin d´étudier les facteurs associés à la survenue des allo-anticorps anti-érythrocytaires. Les variables étudiées étaient: l´âge, le sexe, la gestité chez les femmes, les signes d´hémolyse, le diagnostic, le nombre de poches de concentrés de globules rouges transfusé et le délai de la dernière transfusion reçue. Le seuil de significativité des tests avait été fixé à 5% (p < 0.05). Le consentement éclairé de chaque patient avait été obtenu avant le prélèvement.

## Résultats

**Caractéristiques générales des patients:** nous avons inclus 51 patients dont 26 dans le service d´Hématologie clinique et 25 en Néphrologie. Une prédominance masculine avait été notée avec un sex-ratio à 1,43 (30 hommes pour 21 femmes) et une moyenne d´âge de 44,6 ans ± 17,2 ans. L´âge moyen des patients recrutés dans le service d´Hématologie clinique étaient de 39,34 ans ± 18,66 ans tandis que ceux recrutés dans le service de Néphrologie étaient âgés de 50 ans ± 13,82. La répartition des polytransfusés de sexe féminin montrait que 10 sur les 21 femmes incluses (47,62%) étaient des nulligestes. L´hypertension artérielle (HTA) et le diabète étaient les antécédents médicaux recensés dans respectivement 15,68% et 7,84% des cas. Les antécédents chirurgicaux retrouvés étaient la splénectomie dans 02 cas, une cure herniaire inguinale dans 01 cas et 1 cas d´ hémorroïdectomie. La répartition des patients selon leur diagnostic, montrait que tous les patients recrutés dans le service de Néphrologie étaient suivis pour une insuffisance rénale chronique d´étiologies diverses. Parmi les patients inclus en Hématologie, 09 étaient suivis pour une hémopathie maligne, 06 pour un syndrome drépanocytaire majeur, 04 pour aplasie médullaire (Tableau 1). La répartition des patients en fonction de leur groupe sanguin dans le système sanguin ABO montrait que vingt-sept (27) patients étaient du groupe O (52,95%), 11 du groupe B (21,56%), 09 du groupe A (17,64%) puis 4 patients du groupe AB (7,85%). En ce qui concerne le groupage dans le système RH, Seul le RH1 avait été systématiquement recherché chez tous les patients inclus. Ce qui a permis d´identifier 90,2% de patients RH1 positifs (Tableau 2).

**Tableau 1:** répartition des patients en fonction des étiologies

Diagnostic	Effectif	Fréquence (%)
IRC par néproangiosclérose bénigne	16	31,38
Syndrome drépanocytaire majeur	8	15,7
IRC par néphropathie diabétique	4	7,84
Néphroangiosclérose maligne	4	7,84
Aplasie médullaire	4	7,84
Anémie par infection à VIH	2	3,92
Leucémie myéloïde chronique	2	3,92
Leucémie lymphoïde chronique	2	3,92
Leucémie aiguë lymphoblastique	2	3,92
Syndrome myélodysplasique	2	3,92
Lupus érythémateux systémique	2	3,92
Leucémie aiguë myéloblastique	1	1,96
Hémophilie	1	1,96
Néphropathie secondaire au VIH	1	1,96
Total	51	100

**Tableau 2:** répartition des patients en fonction des groupes sanguins ABO RH1

Groupe sanguin dans le système ABO	RH1	Effectif	Fréquence (%)
O	Positif	25	49,02
	Négatif	2	3,92
B	Positif	10	19,61
	Négatif	1	1,96
A	Positif	8	15,69
	Négatif	1	1,96
AB	Positif	3	5,88
	Négatif	1	1,96
Total		51	100

**Dépistage et identification des allo-anticorps anti-érythrocytaires:** la recherche des anticorps anti-érythrocytaires avait été positive chez sept (07) patients sur les 51 inclus, soit une fréquence de 13,73%. Cependant les allo-anticorps anti-érythrocytaires n´avaient pu être identifiés que chez 3 patients sur les 7. Les spécificités retrouvées étaient: une association anti-RH1 et anti-RH3 chez un des patients, un anticorps isolé anti-LE1 pour le 2^e^ cas, puis l´association anti-RH3 + anti-FY1 pour le dernier cas. Les quatre (04) autres patients présentaient une difficulté d´interprétation de la RAI notamment: deux cas de RAI positifs avec le témoin autologue positif; et deux cas de pan-agglutination dont: un cas de pan-agglutination avec auto-test positif; un cas de pan-agglutination avec auto-test négatif.

### Caractéristiques des patients possédant des allo-anticorps anti érythrocytaires

**Cas 1:** patiente de sexe féminin, âgée de 50 ans, multigeste, multipare, était suivie pour un lupus érythémateux systémique ayant reçu 5 poches de CGR lors de la prise en charge d´une anémie hémolytique sévère. L´hémogramme réalisé montrait une anémie normocytaire régénérative. On ne notait ni d´hémoglobinémie, ni d´hémoglobinurie. La bilirubinémie et le dosage de la lactate déshydrogénase étaient normaux. Les allo-anticorps anti-érythrocytaires identifiés étaient l´anti RH1 et l´anti RH3. Son phénotype étendu était: AB, RH: -1,-2,-3,4,5; KEL: 1,2; JK: -1,2; FY: -1,-2; LE: -1,-2. La transfusion de globules rouges était efficace avec une augmentation de 2,1g/dL d´hémoglobine après une transfusion de 2 poches.

**Cas 2:** patiente âgée de 55 ans, multigeste, multipare, ayant un antécédent d´hypertension artérielle, était suivie dans le service de Néphrologie pour une insuffisance rénale chronique secondaire à une néphroangiosclérose bénigne. Environ 16 poches de CGR lui avaient été transfusées au cours de ses différentes séances de dialyse. Au moment de l´inclusion, l´hémogramme de cette patiente révélait une anémie normocytaire arégénérative. Aucun signe d´hémolyse clinique n´avait été identifié. On ne note ni d´hémoglobinémie, ni d´hémoglobinurie et la bilirubinémie était normale. Cependant le dosage de la lactate déshydrogénase était à 1,13 fois la normale soit 397 UI/L (VN: 90-350 UI/L). Son phénotypage érythrocytaire permet d´identifier: O; RH: 1,-2,3,4,5; KEL: -1,2; JK: 1,-2; FY: -1,-2; LE: -1,-2. L´allo-anticorps anti-érythrocytaire identifié était l´anti-LE1. La transfusion de globules rouges était inefficace avec une augmentation de 0,6g/dL d´hémoglobine après une transfusion d´une poche.

**Cas 3:** patient âgé de 44 ans, de sexe masculin, suivi pour une anémie hémolytique en cours d´exploration au moment de l´étude. L´examen clinique notait un subictère après 28 poches de concentrés de globules rouges. L´anémie était normocytaire régénérative. La recherche des stigmates biologiques avait montré une hémoglobinémie et une discrète hyperbilirubinémie (13,1mg/L) à bilirubine libre (7mg/L). La lactate déshydrogénase était élevée à 1,3 fois la normale soit 463 UI/L (VN: 90-350 UI/L). Son phénotypage érythrocytaire a permet d´identifier B; RH: 1,-2,-3,4,5; KEL: -1,2; JK: 1,-2; FY: -1,-2; LE: -1,-2. La recherche d´agglutinine irrégulière a permis de mettre en évidence une association d´anti-RH3 et d´anti-FY1. La transfusion de globules rouge était inefficace avec une augmentation de 0,4g/dL d´hémoglobine après une transfusion de 2 poches.

**Recherche de facteurs associés:** la présence des allo-anticorps anti-érythrocytaires étaient statistiquement associé à la transfusion de plus de 15 poches de concentrés de globules rouges (p=0,04). Il en est de même pour la multigestité qui était associée de la présence des allo-anticorps anti-érythrocytaire chez les patients de sexe féminin (p=0,0196). Notons que ni l´âge (p=0,371), le sexe (0,355), le diagnostic (0,15) et la présence des signes d´hémolyse (0,06) n´étaient associés à la présence des allo-anticorps anti-érythrocytaires.

## Discussion

Nous avons inclus les polytransfusés dont la dernière transfusion datait de moins de trois mois car le titre d´un anticorps anti-érythrocytaire, identifié à un moment quelconque du traitement transfusionnel, peut diminuer avec le temps [10]. En effet, certains allo-anticorps anti-érythrocytaires pourraient disparaitre après trois mois, notamment les anti-JK [9]. Par ailleurs, tous les patients que nous avons inclus au cours de l´étude avaient reçu au moins deux poches de CGR, comme dans la revue de littérature [4, 5].

La recherche des allo-anticorps anti-érythrocytaires avait été réalisée par le test indirect à l´antiglobuline en milieu de basse force ionique (solution LISS) sur un support de filtration conformément aux recommandations de l´arrêté du 26 avril 2002 du Ministère de la Santé de la République Française modifiant l´arrêté du 26 novembre 1999 relatif à la bonne exécution des analyses de biologie médicale [11]. Cette technique est très sensible, bien standardisée et permet de mettre en évidence l´ensemble des anticorps cliniquement significatifs [9]. Elle a souvent été utilisée par d´autres auteurs dans la littérature [5].

La fréquence de l´allo-immunisation anti-érythrocytaire au sein de notre population d´étude était de 13,73%. Depuis les débuts de la transfusion sanguine, différentes études ont tenté d´estimer la prévalence de l´allo-immunisation post-transfusionnelle. Sur une série malienne de 78 polytransfusés, la fréquence d´allo-immunisation anti-érythrocytaire était de 10,3% [4] et de 3,79% en Inde [12]. Par contre, en Tunisie, une étude réalisée par Amor *et al*. a permis de révéler une prévalence de 17,5% d´allo-immunisation anti-érythrocytaire sur 84 patients hémoglobinopathes [5]. Cependant, ces études sont difficilement comparables du fait de la variabilité des périodes d´études, des méthodes de recueil des données, des pathologies sous-jacentes des receveurs ainsi que de la réglementation concernant les tests obligatoires pré-transfusionnels des pays où se déroulaient chacune des études.

L´identification des allo-anticorps anti-érythocytaires a pu être réalisée chez 3 patients sur les sept (07) dépistés. Deux cas de poly-immunisation ont été identifiés associant 2 types d´allo-anticorps (association anti-RH1+ anti RH3 et association d´anti-RH3 + anti FY1) et un cas d´anticorps anti-érythrocytaire isolé (anti-LE1). Trois de ces allo-anticorps identifiés correspondaient à des antigènes réputés immunogènes. Selon la littérature, les antigènes de groupes sanguins les plus immunogènes, sont par ordre décroissant d´immunogénicité les antigènes: RH1, KEL1, RH3, RH4, FY1 et JK1 [1]. L´anti-LE1 identifié chez l´un des patients au cours de notre étude, avait été retrouvé au cours d´une autre étude réalisée chez les gestantes béninoises [13]. D´autres auteurs ont rapporté des spécificités similaires aux nôtres [14, 15].

Par ailleurs, les quatre (04) cas de RAI positive d´interprétation difficile, étaient des cas de témoin autologue positif et de pan-agglutination. Les cas de pan-agglutination étaient associés pour l´un à un témoin autologue positif et pour l´autre à un témoin autologue négatif. Un témoin autologue positif pourrait traduire la présence d´un auto-anticorps anti-érythrocytaire dont la preuve serait apportée grâce à des épreuves d´auto-absorption de l´anticorps associé à une RAI réalisée sur le sérum adsorbé [9]. Ce qui n´a pas pu être réalisé au cours de notre étude. Cependant un conflit par incompatibilité transfusionnelle n´est pas formellement écarté car tous les patients recrutés avaient reçu au moins une poche de concentrés de globules rouges au cours des 3 derniers mois.

Quant à l´exploration des cas de pan-agglutination, elle n´avait pas pu être réalisée au cours de notre étude pour des raisons de plateau technique limité. Cette étude a permis de noter que l´allo-immunisation était plus fréquente chez les patients ayant reçu plus de 15 poches (p=0,04). Ce résultat se rapproche de celui de Rosse *et al*. [15] qui avait montré que la fréquence de l´allo-immunisation anti-érythrocytaire augmentait à partir de 12 poches de CGR reçues dans une population d´hémoglobinopathes polytransfusés. Comme la plupart des études, l´âge, le sexe, le diagnostic et le service de provenance n´étaient pas associée à l´apparition des allo-anticorps anti-érythrocytaires [16]. Cependant l´effectif de notre population est faible et la reprise de l´étude sur une population d´étude plus importante pourra permettre d´étudier au mieux les liens qui existeraient entre l´allo-immunisation anti-érythrocytaire et les différentes variables étudiées.

## Conclusion

L´allo-immunisation anti-érythrocytaire post-transfusionnelle représente une complication immunologique très fréquente de la thérapie transfusionnelle au Bénin. Il urge de mettre en place la recherche systématique d´agglutinine irrégulière en période pré et post transfusionnelle particulièrement chez les polytransfusés.

### Etat des connaissances sur le sujet

- La transfusion sanguine est un acte médical qui s´associe à la survenue de plusieurs complications notamment celles immunologiques; ce risque est plus élevé chez les polytransfusés pouvant aller de 9,8% à 28% selon les auteurs;
- Au Bénin, un dépistage des allo-anticorps anti-érythrocytaires n´est pas systématiquement réalisé chez les polytransfusés.

### Contribution de notre étude à la connaissance

- Cette étude a permis de déterminer la prévalence de l´allo-immunisation anti-érythrocytaire chez 51 polytransfusés suivis au CNHU-HKM de Cotonou;
- Les allo-anticorps anti-érythrocytaires ont été dépistés chez 7 patients soit une prévalence de 13,73%. Les allo-anticorps retrouvés étaient: l´anti-RH1, l´anti-RH3, l´anti-LE1 et l´anti-FY1.
